# Enhancing post-diagnostic care in Australian memory clinics: Health professionals’ insights into current practices, barriers and facilitators, and desirable support

**DOI:** 10.1177/14713012231213419

**Published:** 2023-11-24

**Authors:** Slađana Pavković, Lynette Ruth Goldberg, Maree Farrow, Jane Alty, Melissa Abela, Sharon Naismith, Perminder Sachdev, Lee-Fay Low

**Affiliations:** Wicking Dementia Research and Education Centre, University of Tasmania, 60119 College of Health and Medicine, Australia; Wicking Dementia Research and Education Centre, 60119 University of Tasmania, College of Health and Medicine, Australia; 3925 Wicking Dementia Research and Education Centre, University of Tasmania, College of Health and Medicine, Australia; 4334 The University of Sydney, School of Psychology, Australia; 7800 Centre for Healthy Brain Ageing Neuropsychiatric Institute Euroa Centre, Prince of Wales Hospital, Australia; 4538 University of Sydney Faculty of Health Sciences, Australia

**Keywords:** dementia, diagnosis, memory clinics, post-diagnostic support, health professionals, Australia

## Abstract

**Introduction:**

Providing integrated and evidence-based support to individuals and families following a diagnosis of dementia is essential in order to optimise their quality of life and assist them to live well. Memory clinics provide multidisciplinary services specialising in the assessment and post-diagnostic treatment of people with dementia. This study sought to identify current practices, barriers and facilitators to provision of postdiagnostic support and to obtain health professionals’ opinion of ideal post-diagnostic support to be offered in Australian memory clinics.

**Methodology:**

This was a cross-sectional qualitative exploratory study. Data was collected from health professionals familiar with the process of diagnosis and post-diagnostic support through two expert panel meetings (*n* = 22). In addition, 5 focus groups (*n* = 22) were conducted including health professionals who are employed in Australian memory clinics. Data was collected between October 2020 and November 2021. Reflexive thematic analysis was undertaken.

**Results:**

Seven themes and three subthemes were identified under the three topics: Current Practices, Barriers and Facilitators, and Desirable Support. Themes relating to Current Practices were: Tailored Communication and feedback about diagnosis; Prescription of medications and follow-up; and Referrals to health and community services. Themes relating to Barriers and Facilitators were: The structure of the current system; Lack of funding; Lack of resources; Call for government investment. Themes relating to Desirable support were: A key/single point of support; Cognitive interventions; and Counselling and education.

**Conclusion:**

Post-diagnostic support in Australian memory clinics focused primarily on ensuring people understood their diagnosis, information about postdiagnostic support was provided, and dementia medications were prescribed. There were notable differences in practices in metropolitan compared to regional areas. A key concern was the need for increased funding, particularly to support the establishment of a single point of contact to facilitate continuity of care.

## Background

There are many potential benefits to an early dementia diagnosis. These include receiving treatments that may potentially slow functional decline, providing opportunities to make decisions about the future, and enabling participation in clinical trials and other research (Epperly et al., 2017; Krolak-Salmon et al., 2017; Porteri et al., 2017; Robinson et al., 2015). However, the consequences of an early diagnosis of dementia are not always positive. The fear of an uncertain future, and the risk of losing one’s autonomy, including the ability to make decisions and act independently are paramount (Gauthier et al., 2013; Xanthopoulou et al., 2019). This may lead to anxiety, depression, and a sense of hopelessness (Dubois et al., 2016; Morgan, 2021). Further, a diagnosis of dementia impacts on family members who anticipate needing to take on a caring role (Braun et al., 2009; de Vugt & Verhey, 2013; Pozzebon et al., 2016).

Providing integrated and evidence-based support to individuals and families following a diagnosis of dementia is essential and advocated in the 2022 World Alzheimer’s Report (Gauthier et al., 2022). This integrated and evidence-based support is commonly referred to as post-diagnostic support. Overall, post-diagnostic support is defined as *treatment* (pharmacological and non-pharmacological interventions) and *support programs* (e.g., written information, counselling, education, peer support groups, dementia advisory services), *after dementia diagnosis, for people with dementia and their care partners* (Dodd et al., 2018; Kelly & Innes, 2016; O’Shea et al., 2018). Post-diagnostic support aims to assist people with dementia to live well and optimise quality of life through alleviation of the challenges of dementia diagnosis and dementia progression (Aldridge et al., 2019; Cations et al., 2019; Copland et al., 2018).

In Australia, the importance of providing post-diagnostic support is recognised in the *National Framework for Action on Dementia 2015–2019* (Australian Government Department of Health, 2015). Australia adopted the *Global Action Plan on Public Health Response to Dementia 2017–2025* in response to the call from the World Health Organization (2017). This Global Action Plan established the need for care after diagnosis and support for care partners as public health priorities. Prior to Australia’s adoption of the Global Action Plan, post-diagnostic support was frequently discussed by Australian health professionals, policy makers and lived experience experts, and these discussions resulted in numerous recommendations in pivotal guidelines for health professionals. For example, the *Clinical Practice Guidelines and Principles of Care for People with Dementia* (Guideline Adaptation Committee, 2016) established the importance of post-diagnostic services in Australia, including information and advice about future care. Following the adoption of the Global Action Plan, the first Australian *Memory and Cognition Clinic Guidelines* (Section 13) provided recommendations for post-diagnostic support in Australian memory clinics (Australian Dementia Network, 2021). Subsequently, the Final Report of the Australian Royal Commission into Quality and Safety in Aged Care recommended a “comprehensive, clear and accessible dementia support pathway” (Recommendation 15, p. 220) for people living with dementia and their care partners, to be implemented from January 2023 (Pagone & Briggs, 2021).

Memory clinics (MCs) provide multidisciplinary services specialising in the assessment and post-diagnostic treatment of people with dementia (Naismith et al., 2022). In Australia, memory clinics are encouraged to register and become part of the Australian Dementia Network (ADNeT). ADNeT is an initiative funded by the Australian National Health and Medical Research Council that aims to improve and harmonise the process of diagnosis, facilitate access to memory clinics across the country and improve post-diagnostic care (Australian Dementia Network, 2021). In Australia, publicly operated memory clinics are funded primarily to provide a dementia diagnosis; their provision of post-diagnostic intervention and support appears limited (Naismith et al., 2022). The first ADNeT Clinical Quality Registry (CQR) Report, completed in 2020, revealed that out of 40 registered clinics, 29 were in metropolitan areas and 11 in regional areas; 18 registered clinics were private and 22 public (Ward et al., 2021). The number of registered clinics has now almost doubled (ADNeT, 2023). Seventy-four memory clinics are currently registered on the ADNeT CQR and more than 150 clinics are mapped on the ADNeT website (ADNeT, 2023). The process of accreditation is the next step for Australian Memory clinics, which is based on the UK’s example, to ensure that services provided meet established quality standards (ADNet, 2023a).

In addition, memory clinics in the UK are able to link clients with post-diagnostic supports through a single service, such as “link worker”, “admiral nurse” or “dementia advisor” similarly, in the Netherlands, a “care navigator” or “case manager” can link client’s to post-diagnostic supports (Alzheimer’s Scottland, 2023; Dementia Concern, n. d; Giebel et al., 2021; Gruters et al., 2019; Vroomen et al., 2012). Further, the number of memory clinics that provide cognitive stimulation therapy or some form of psychosocial intervention in the UK is growing (Cook et al., 2020; Eikelboom et al., 2022; Royal College of Psychiatrists, 2022). In regards of cognitive intervention implemented in memory clinics, the UK model influence the first steps in Australia. Indeed, the implementation of cognitive interventions is a current pilot project included 1-2 memory clinics per each state (ADNet, 2023b).

In Australia, two surveys have described the limited provision of post-diagnostic support in both public and private memory clinics (Mehrani et al., 2021; Naismith et al., 2022). This is in sharp contrast to the need for such support (Gauthier et al., 2022; Steiner et al., 2020). Of 150 health-professionals from 90 memory clinics across Australia, 31% reported that they provided one session of post-diagnostic support including either psychoeducation, involvement of a family member or a guide to rehabilitation. The remaining 69% did not report provision of any post-diagnostic services (Mehrani et al., 2021). In the survey by Naismith et al. (2022), 55 out of 100 respondents from 60 memory clinics commented on their provision of pharmacological care (94%), psychoeducation or counselling (40%), and memory strategy training to support everyday life (20%). The remaining 45 participants did not report providing any post-diagnostic support services.

The purpose of the current study was to understand the reasons for this limited post-diagnostic support and enrich our understanding of current and ideal support.

Three questions were posed:

(1) Do memory clinics offer post-diagnostic support and, if so, what support do they offer?
(2) What are the barriers and facilitators to providing post diagnostic support in Australian memory clinics?
(3) What support do healthcare professionals think should ideally be offered by memory clinics in Australia?

## Methodology

This was a cross-sectional qualitative exploratory study, specifically designed to address the gap in contextual evidence due to the quantitative nature of the previous studies. Qualitative methodologies are ideal to explore, inform, and process key contextual factors (Creswell & Creswell, 2018).

The study included data from two expert panel meetings and five focus groups, all featuring health professionals working in Australian memory clinics and involved in the process of dementia diagnosis.

This study was approved by the Human Research Ethics Committee of the University of New South Wells reference number HC200394 and the University of Tasmania (HC23946). All participants provided written informed consent.

### Expert panel meetings

Participants were recruited via the ADNeT MC network and included an array of professionals from varied clinical settings (e.g., regional vs. metropolitan, public vs. private) and Australian states/territories (Table 1). Participants were selected based on their expertise in dementia diagnosis, clinical experience and their reputation in dementia assessment and post-diagnostic support. From 28 invited healthcare professionals, 22 agreed to participate.

**Table 1.** Background of participants, type of the clinics and locations.

	Panel meetings *n* = 2 meetings; 22 participants	Focus group meetings *n* = 5 meetings; 22 participants
**Health professional role**		
Geriatrician	4	8
Neurologist	3	1
Neuroscientist	2	0
Occupational therapist	2	1
Nurse practitioner	2	3
Registered nurse	1	3
Clinical nurse consultant	0	1
Social worker	1	0
Psychiatrist	2	1
Clinical psychologist	1	0
Speech pathologist	1	1
Neuropsychologist	1	2
Dietician	1	0
General practitioner	1	1
**Type of clinics**		
Private	1	3
Public	11	10
Hybrid	1	1
**Locations**		
Metropolitan	12	9
Regional	1	5
**States**		
New South Wales (NSW)	7	3
Victoria (VIC)	4	7
Western Australia (WA)	3	3
South Australia (SA)	3	2
Queensland (QLD)	3	3
Tasmania (TAS)	1	3
Northern Territory (NT)	1	0

Two 3-h meetings were conducted between October and December 2020, using an on-line Zoom platform. The meetings formed part of a Delphi study to develop National Memory Clinic Guidelines (Australian Dementia Network, 2021). The expert panel meetings’ topics reflected the results of the survey conducted as the first phase of the Delphi study and focused on current and ideal practices relating to the process of diagnosis, diagnostic tools and the communication of a dementia diagnosis and follow-up (Naismith et al., 2022). The topics were sent to the participants seven days before the first meeting. The same participants were invited for the second meeting. Two participants could not attend but provided written feedback which was included in the analysis. The topics discussed in the second expert panel meeting were case conferences, follow-up, referrals to other services and criteria for accreditation of memory clinics. Both meetings were facilitated by experts with clinical and research expertise.

### Focus groups

Twenty-one additional health professionals who were employed in memory clinics participated in five focus groups (4-5 participants/FG) between June and November 2021. As in the expert panel, participants represented different professions and locations (Table 1). Each focus group lasted 60–90 min, was conducted on-line via the Zoom platform, and facilitated by the first author who has qualifications and experience as a dementia care specialist. Participants were invited through ADNet MC mailing lists and newsletters.

Some clinics had more than one representative participant, but each participant attended only one focus group. The focus group guide addressed the same topics discussed at the expert panel meetings. However, participants from focus groups had increased time to discuss the questions, and seven specific questions about post-diagnostic support were asked *1) how do you communicate information about diagnosis? 2) what type of information (written or verbal) do you provide? 3) do you provide information about post-diagnostic resources? 4) what post-diagnostic services and treatments are provided by your memory clinics*? *5) what are the barriers to the provision of post diagnostic support? 6) what are feasible strategies to overcome these barriers?* 7) *what post-diagnostic services could be provided in a memory clinic from an ideal-world perspective*?.

### Data collection and analysis

The data obtained from participants in expert panel meetings and focus groups were combined, following accepted protocol to achieve data richness (Lambert & Loiselle, 2008). The combination of expert panel meeting and focus group data, two qualitative methods, led by different facilitators exploring the same phenomenon of interest, enabled different interactions, contributing to data trustworthiness and validity of the study.

Transcripts of expert panel and focus group meetings were obtained via the Zoom transcription application and corrected for accuracy (audio recordings were compared with written transcripts and edited by the first author). Following confirmation of the transcripts, NVivo (qualitative data analysis software) was used to analyse the data and generate codes (Creswell & Creswell, 2018). Reflexive thematic analysis was conducted using the six-stage analysis proposed by Braun and Clarke (2006) and their later revision that optimised data from different sources enabling a flexible, explorative, and iterative analysis (Braun & Clarke, 2019). Two researchers independently coded the transcripts to aid credibility and trustworthiness of findings. The description of codes and final codes were discussed between the first and senior authors (SP & L-FL). The themes and subthemes then were generated by these authors. Findings were discussed regularly by all authors and revised, which is a regular practice of reflexive thematic analysis (Braun & Clarke, 2019).

## Findings

### Participants

Table 1 presents the qualification/speciality of the participants, and the number, type, location, and state/territory of the memory clinics in which they worked. Most participants worked in public memory clinics in metropolitan areas of NSW and VIC. Of the 44 participants, 12 were geriatricians and 10 were nurses. Other medical specialities were neurologist (4), psychiatrist (3), and General Practitioner (2). The remaining 13 participants represented other health specialties: occupational therapy (3), speech pathology (2), clinical psychology (1), neuropsychology (3), dietetics (1), social work (1), and neuroscience (2). From 44 participants, 8 participants attended both expert panel meetings and focus groups.

### Thematic analysis

**Figure 1.**
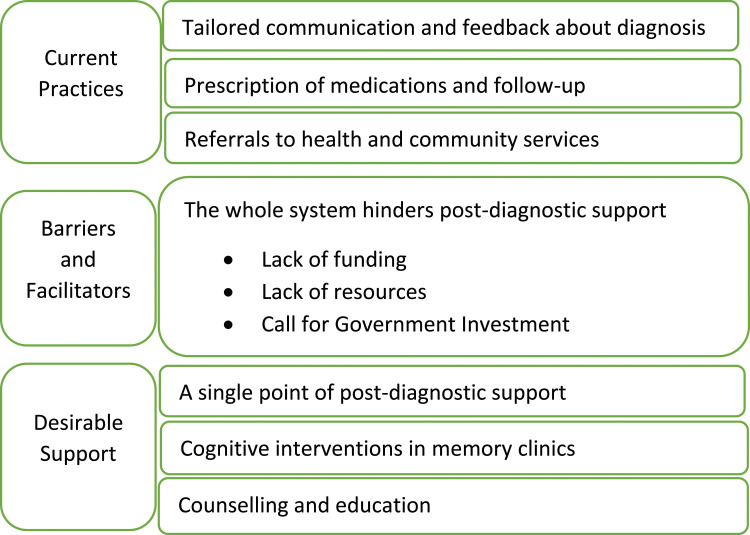
Topics: current practices, desirable support, and barriers and facilitators to post-diagnostic support in Australian memory clinics and the themes relating to each of the topics.

The identified themes and subthemes in response to the three research questions are illustrated in Figure 1

. The responses to the first question “Do memory clinics offer post diagnostic support? If so, what support do they offer?” are summarised and presented under the topic of *Current Practices.* Responses to the second question “What are the barriers and facilitators to provide the needed post diagnostic support in Australian memory clinics?” are summarised under the topic of *Barriers and Facilitators*. Responses to the third question “What post diagnostic support should ideally be offered in memory clinics?” are summarised and presented under the topic of *Desirable Support*.

## Current practices

### Tailored communication and feedback about the diagnosis

The initial appointment that served to communicate the diagnosis and obtain post-diagnostic support was termed a “feedback” session. All clinics organised a feedback session soon after all tests and assessments were completed.

**“**So, we do it after all the investigations have been done and we actually give the written information and explain the diagnosis” **(geriatrician, EPM)**

Participants stated that during this feedback session they addressed comments to both the person with dementia and care partners and tried to tailor the communication and gradually disclose the diagnosis based on whether the person wanted to know and how much they wanted to know, for instance:

“You need to know what the diagnosis means for them, and you can usually pick up on some nonverbal cues around that as well, both from the family member and that person themselves. Sometimes the family member [is] breaking down in the background, so you need to deal with that. You need to then spend time talking about their strengths and acknowledging that this diagnosis doesn't change who they've been. It means there's an explanation for their problems, and you know building up their self-esteem” (**occupational therapist, FG**)”.

All memory clinics’ staff reported that they provide written information relating to diagnosis, to enable their clients to remember all relevant information after the appointment.

“We provide them with a folder of written information to take away as well as handwritten information about the actual discussion on the day of the feedback session” **(speech pathologist, FG)**

Clinics differed in the written information provided relating to post-diagnostic treatments. Some clinics included extensive information about driving, legal matters such as advance care planning, and strategies to optimise everyday life. Most clinics included information about Dementia Australia or provided leaflets or help-sheets from Dementia Australia which included helpline and Web site details.

“Oh, we have a whole bunch of information brochures from Dementia Australia which we give to the clients.” (**geriatrician, FG**)

Support for care partners was discussed through the provision of information about Dementia Australia services and Care Gateway, a service that provides general information for all carers in Australia and care respite services. For example,

“And we also … like to have a bit of a look at the carer's needs as well. So, you know, we asked them ‘are you aware there's something [called] a Carer's Gateway”. We spend some time to explain this and suggest [they] call the service.” **(neuropsychologist, FG).**

### Prescription of medications and follow-up

All healthcare professionals discussed prescribed medications relating to dementia. If people with dementia wanted to start immediately with medications (e.g., cholinesterase inhibitors, memantine), the doctor in the clinic prescribed the medications. Some public clinics provided one follow-up session to check tolerability of medications.

“We want to check tolerability and effect of medications, so we schedule another appointment for clients who start the treatment” **(occupational therapist, EPM).**

If the dementia diagnosis had not yet been confirmed, or if the person was diagnosed with mild cognitive impairment (MCI), public clinics usually provided follow-up. At all public clinics, individuals with a confirmed diagnosis of dementia were referred to their GP for ongoing follow-up. People with dementia attending private clinics received regular and tailored follow-up by the medical professionals there, reflecting the advantage of more time for personalised care than was available at public clinics. For example,

“Number one, you get continuity as a patient of private clinic. Number two is I will get them back for however many reviews they need. I talk to them and carers and shape their care according to their needs… When I started in private practice, I recognized that when a GP sends a patient to a specialist what they want from that specialist is not just the diagnosis and management plan, they actually want you to carry out the management plan.” **(geriatrician, FG).**

Participants from both private and public memory clinics emphasised the importance of continuity of care for their clients. However, opportunities to provide post-diagnostic support at public clinics appeared limited due to their focus on providing diagnostic support.

### Referrals to health and community services

As they were limited in their capacity to provide post-diagnostic support, participants from public memory clinics referred their clients to other local services, for example allied health, that were co-located or located in a nearby hospital.

“Our memory clinic is working closely with allied health teams in local community health services” (**occupational therapist, EPM).**

Participants from the hospital-based memory clinics could offer allied health services available through the hospital:

“Speech therapy or occupational therapy … are usually in the local hospital where I work. It’s quite good in that regard for the local people in the area, because they could easily have what they need” **(registered nurse, FG).**

However, community-based clinics were often not linked to community-based allied health services or they did not exist in the area where the memory clinics operated.

Referrals were also made to peer support groups, Aged Care Assessment, the *Living with Dementia Program* provided by Dementia Australia, or local services such as an exercise group, depending on clinician knowledge of the services and their availability in the local area. One participant who worked in both urban and regional public memory clinics, commented:

“It then depends on where I am. We actually have good access to exercise physiologists down -they've [patients] got to go 150 kilometres to get a neuropsychological assessment, but we do have an exercise physiologist who understands about dementia.” **(geriatrician, FG)**

Participants at private clinics referred their clients to non-pharmacological services in accordance with the clients’ needs as well as participants’ knowledge about available local services. At times, participants’ desire to address their clients’ needs appeared to reflect their own opinions. As an example, one participant who represented a private clinic added:

**“**I personally don't refer them for a driving assessment until such time as I've got them on the drugs, I've got them as well as possible, because I want to maximize the chance of passing the driving assessment. I get quite a few patients from public clinics who come and see me after they have been told to stop driving. They're furious because they never got a chance to really do the best with the driving assessment… Referrals really depend on the patient and the situation” **(geriatrician, FG)**

Professionals at private memory clinics were able to spend more time following-up patients as required, and supporting them through difficult aspects after diagnosis such as driving and decision making. This was not possible within the constraints of most public memory clinics.

Participants from regional clinics stated that they did not have many local services and thus limited opportunities for referrals. However, referrals were made to My Aged Care (national gateway for aged care services) and, when available, local Dementia Outreach teams for home support and assessments.

## Barriers and facilitators

### The whole system hinders post-diagnostic support

Participants focused on multiple constraints relating to the health system and clinic organisation and concluded that the current healthcare system does not facilitate integrated support for people with dementia and their care partners. All participants agreed that the health system was a major barrier to providing post-diagnostic support in Australian memory clinics.

#### Lack of funding

A chief concern was lack of funding. This limited the capacity of public and private clinics to provide post-diagnostic support. Funding affected the number of staff, clinic operating days and times, and the comprehensiveness of available resources that could be provided onsite and in local areas. Participants working in public clinics felt the lack of funding most acutely. Many participants pointed out that due to the lack of funding they had to discontinue a service.

“When we've had opportunities to do a little bit extra with people, we were able to make some really great differences, but unfortunately that bucket of money doesn't tend to hang around very long. It is stupid. We have tried something that works brilliantly, and then the funding disappears. I mean this is the system. It is a political issue” **(geriatrician, FG)**.

Some participants endorsed the notion of a key support person, but funding was an issue, so they suggested:

“A dementia key worker is necessary for all memory clinics but from Dementia Australia - with funding - would be very useful” (**neurologist, EPM**).

#### Lack of resources

Reflecting concerns about funding, participants commented on the impact on information about local services, including how information was presented. A few participants from public clinics stated that they have a structured template with post-diagnostic information about managing everyday life, obtaining driving assessment and accessing resources through Dementia Australia. However, the majority claimed that they never had such a template, and they lacked time and staff that could enable creation of such a resource. For example:

“It's about knowing what services are available in your area. You need somebody who's across the whole system. It's hard to stay on top of that, because things are always changing, a new thing is popping up” **(general practitioner, FG)**.

A particular concern for participants working in regional areas was the lack of local services, including being able to refer people to Dementia Australia.

“I guess, away from the public funding, a constraint is that we don't have a lot [of services here] at all” **(geriatrician, FG)**.

An overall concern remained about the limited support for post-diagnostic care with available resources directed more to assessment; reflecting the focus on assessment, others commented on the limited resources and need for referral back to GPs:

“We give a very comprehensive letter to the GPs afterwards, and they have to carry the full weight of what happens next. We don't have resources at the current time” (**nurse practitioner, EPM**).

#### Call for government investment

The idea of developing successful communication with the Department of Health was mentioned to facilitate continuation of care and enable ongoing support with navigation through the system, for example:

“Regarding the dementia navigator I think that's a really good idea, but there's no funding so technically the person, the medical person, or the helpers and who sees the patient, has to be the dementia navigator as well as the treating physician or psychiatrist. This is how the system works. We need to talk more with the Department of Health” **(geriatrician, FG).**

Criticising the organisation of the Australian health system, several participants compared the system in Australia with the UK.

“I mean you'd have to rebuild the health system in Australia to have something like UK [where] it is all one entity and under National Health Service **(geriatrician, FG)”**

Overall, participants did not feel that they had capacity to deal with the system, but they thought that the issues with the health system could be addressed only if the Australian government was involved and ready to invest in the change.

“Federal and state governments should consider baby boomers, and from there they can activate the services. It's a big dream for all of us over here, but something which has to be prepared at high levels” **(geriatrician, FG).**

Only one participant stated that he was involved in consultation with the Department of Health and Aged care, but he was disappointed with the current outcome.

## Desirable support

Expert panel meetings’ participants reluctantly discussed ideal post-diagnostic support because of the barriers they perceived to providing services after diagnosis. In the Focus groups, the facilitator reframed the topic from ideal to desirable post-diagnostic support, and participants appeared more comfortable with discussing this amended topic.

### A single point of post-diagnostic support

In their discussions, participants focused extensively on the need for a single point of support. This key support person would be responsible for linking people with dementia and their care partners with other support services, answering questions about dementia, and suggesting strategies to optimise daily life.

“I know that with other diseases … there is a specific person that they get in contact with, and then they guide them through the rest of the process for the subsequent years. I think that's a really ideal service for memory clinics. To have continuity is great” **(general practitioner, FG)**.

Participants used different titles for that key role such as navigator, coordinator, and case manager. The common phrases used in describing this important role were “person who explains the system”, “person who coordinates their journey”, “person who will navigate them through the services”. Participants differed in their opinions about the setting in which the key person should be based. Some argued for a position in the memory clinics; others thought that being based in primary care or community or a non-profit organisation would be more cost-effective.

“We just want that one person. Because what they are doing is trying to navigate complex health support services. They (people with dementia and care partners) just don’t know what to do next and how to access a service. It does not matter where this person sits - in memory clinics, a community organisation or in Dementia Australia” **(neurologist, FG).**

### Cognitive interventions in memory clinics

Participants identified different types of cognitive interventions that could be provided in memory clinics such as cognitive rehabilitation, memory strategy training, computerised training, reablement therapy, and cognitive stimulation therapy. A participant who provided cognitive interventions in a private practice shared his experience about delivering cognitive rehabilitation:

“It's rewarding, it's nice to see that people seem to benefit. I tend to assess people before I start a course of an intervention broadly classified as cognitive rehabilitation with a goal setting instrument and some other general cognitive and emotional measures and after 12 sessions, I assess them again and I can see improvement. **(neuropsychologist, FG)**

Many participants agreed that if memory clinics could not provide the needed interventions, staff at the clinics were responsible for facilitating access to these interventions at other sites.

“Cognitive rehabilitation and memory training could be provided in memory clinics, or we can refer. We have worked with our community rehab team to upskill them in cognitive rehabilitation and memory training” (**occupational therapist, FG**)

Participants expressed regret that cognitive interventions were generally not available in Australia and agreed that memory clinics would be appropriate places for integrating and delivering this type of intervention.

### Counselling and education sessions

Participants emphasised the importance of sessions for counselling and education about dementia and the need to initiate these services immediately after diagnosis. Participants felt that offering these sessions at the memory clinics would be ideal. However, they agreed that the value of such services would not be significantly diminished if they were provided at community or primary health care locations. Participants pointed out that a need to talk to someone after the diagnosis was identified in the research as essential and their personal experience confirmed this:

“An ideal service would be counselling, they need to talk, after we communicate with them and disclose the diagnosis. It was suggested in the literature, and we know that” (**nurse practitioner, FG**).

## Discussion

This study presents the views of two groups of health professionals in Australia regarding current post-diagnostic support in Australian memory clinics. All were experienced in dementia assessment and care and most worked in memory clinics. This study is important as there is little published information about post-diagnostic support in Australia and this is the first study to provide contextual information about current practices, and barriers and facilitators regarding post diagnostic support in Australian memory clinics. In addition, this study presents ideas about desirable support after diagnosis in memory clinics and highlights the key issue of establishing an immediate and single point of support after diagnosis. This study enriches the limited data about the services offered by memory clinics internationally.

### Current practices in Australian memory clinics

All clinics offered diagnostic services, but post-diagnostic support focused primarily on ensuring people understood their diagnosis. Information about post-diagnostic support was provided and dementia medications were prescribed. People attending public memory clinics were generally offered one feedback session where they were provided with information to clarify their diagnosis and identify available post-diagnostic services. The practice of using one feedback session in Australian publicly funded memory clinics appears similar to clinics in England, the Netherlands and Canada (Chrysanthaki et al., 2017; Lee et al., 2019; Visser et al., 2019, 2020).

Another post-diagnostic practice common to Australian and international memory clinics was the prescription of medications, primarily cholinesterase inhibitors (Chrysanthaki et al., 2017; Pennington et al., 2018). In public Australian memory clinics, the discussion and management of prescribed medications was generally limited to the initial feedback session. Adults with dementia attending public memory clinics were referred to their GPs for ongoing care and post-diagnostic support, but the caveat is that many GPs do not feel comfortable working with adults with dementia and have a limited understanding of available community resources for post-diagnostic support (Casey et al., 2020; Wang et al., 2020).

In addition to more frequent and tailored care, adults attending private memory clinics in Australia had better access to onsite memory specialist services, which led to continuation of care. This adds to the quantitative survey findings reported by Naismith et al. (2022) whereby private clinics had shorter wait times and more follow up than public clinics. The Memory and Cognition Clinic Guidelines (Australian Dementia Network, 2021) recommend that follow-up needs to be offered according to the client’s needs, and memory clinics need to evaluate each client’s progression and implementation of the care plan within 12 months of diagnosis. The implication is that funding for public memory clinics needs to be extended by Australian state and commonwealth governments beyond the current focus on assessment and diagnosis purposes. One of the identified linked services was referral to an exercise physiologist. Since this service is often a component of Allied Health Services, along with speech pathology, occupational therapy, and physiotherapy, this may increase the opportunity for the referrals to evidence-based activity to facilitate cognitive function for people attending memory clinics (Huang et al., 2022; Sexton et al., 2016)

Differences in practices were apparent between metropolitan and regional memory clinics. As expected, there were more services available in metropolitan compared to regional areas, and thus more opportunities for referral for post-diagnostic specialist support. Memory clinics in regional areas were constrained by the limited post-diagnostic services available in the community, which is a well-recognised disparity in Australia (Van Gaans & Dent, 2018). People with dementia and their care partners living in regional areas experience substantial challenges in accessing services (Arsenault-Lapierre et al., 2023; Dawson et al., 2017; Gardiner et al., 2019; Gibson et al., 2019). Similar challenges accessing post-diagnostic services in regional areas have been reported in England, the Netherlands and Canada (Giebel et al., 2021; Gridley et al., 2019; Lee et al., 2019; Lee et al., 2019a).

### Barriers and facilitators

The primary barrier raised by participants, particularly those from public memory clinics was inadequate funding. Participants in this study viewed this funding barrier as a reflection of the siloed rather than integrated Australian health system. This issue is not unique to Australia. In the Netherlands and UK, funding and the organisation of the health system have been identified as two main barriers to integrated post-diagnostic support (Bamford et al., 2021; Giebel et al., 2021; Wheatley et al., 2021). The concern about funding as an issue that needs to be addressed to enable an improvement in organisation, services, and continuation of care in memory clinics confirmed findings from the two previous Australian studies (Mehrani et al., 2021; Naismith et al., 2022).

The integrated model of care suggested by Low et al. (2023) provides a valuable guide for post-diagnostic support that could be used in memory clinics until the changes proposed in this study are implemented. It is clear that public metropolitan clinics in Australia are developing an integrated model of care using local resources. The problem remains unresolved with regional clinics that lack local resources, although there is an example of a primary care led memory clinic in regional Victoria (Australia) that has shown effective implementation of post diagnostic continuity of care (Disler et al., 2022). The model by Disler and colleagues (2022) provides additional education of GPs and nursing staff along with access to specialist advice and collaboration with secondary care. These components, some provided through telehealth, have facilitated diagnosis, follow-up, and linkage to available local services. Similar models of collaboration between primary care, memory clinics, community partners/agencies and family care also have been implemented in Singapore (Lai et al., 2019).

### Desirable post-diagnostic support

All participants emphasised the need for a single point of support for people with dementia and their care partners. A similar point/key support person is embedded in the integrated model of care provided in multidisciplinary services in the Netherlands under the title of “care navigator” (Iliffe et al., 2019; Vroomen et al., 2012). Key support person models that operate in the UK show numerous variations, but the main characteristic is that the key support person is the crucial factor in effective support following diagnosis (Harper, 2019). In Australia, the model of dementia key support worker was trialled for people with young-onset dementia (onset prior to 65 years of age), which was provided by Dementia Australia with government funding (Westera & Fildes, 2016). In 2019, the model was moved to the disability system and funded through the National Disability Insurance Scheme (NDIS) with more focus on home and community support services and less on person-centred dementia support (Cations et al., 2022).

In the current study, participants focused on the need to provide cognitive interventions which to date have not generally been offered in Australian memory clinics. Participants highlighted that computerised cognitive training, cognitive rehabilitation, reablement or any type of evidence-based cognitive intervention would be beneficial for people with dementia and their care partners. Currently, the Australian government’s Medicare Benefits Schedule (MBS) does not include cognitive interventions; therefore, there is no funding to provide cognitive interventions by neuropsychologists or other allied health professionals (Australian Government, 2022). There is strong evidence regarding the effectiveness of strategy based cognitive training (Mowszowski et al., 2016), computerised cognitive training (Hill et al., 2017) and various cognitive interventions for people living with dementia (Bahar-Fuchs et al., 2020), and the UK experience supports the implementation of such interventions in Australian memory clinics (Cook et al., 2020; Royal College of Psychiatrists, 2022).

Counselling and education sessions were also identified as desirable post-diagnostic services to be offered within memory clinics. In England and Wales psychoeducation is provided in memory clinics, but it is less common than Cognitive Stimulation Therapy (CST) (Royal College of Psychiatrists, 2022). The situation in other parts of the UK seems similar to Australia. For example, in the whole of Ireland, there is only one dementia counselling service that operates one day a week (Morgan, 2021). In Ireland there are 25 memory clinics, and some provide counselling, education, psychoeducation or CST (Pierce et al., 2019). Frequently, post-diagnostic services are provided through a dementia nurse specialist (Gibb & Begley, 2023), which confirms the need for a key contact to drive continuity of care for post-diagnostic services in Australia.

### Limitations and strengths of this study

The majority of participants represented metropolitan and public memory clinics and were from NSW and Victoria. Consequently, the finding form this study may not be generalisable to entire network on memory clinics. The first author (SP) previously worked as a dementia advisor. Therefore, inadvertent bias in the facilitation of the focus groups and interpretation of data is possible, but through the lens of qualitative research this is a strength of this study (Manohar et al., 2018). One of the limitations of this study is that health professionals who participated in focus groups and expert panel meetings have not had an opportunity to give the feedback on thematic analysis. However, several researchers, who were involved in thematic analysis, are health professionals such as a neurologist, speech pathologist, exercise physiologist and psychologist. This contributes to the higher reflexivity with the active involvement of other co-authors who are specialists in other areas of dementia care.

The eight participants were involved in both expert panel meetings and focus groups, therefore they were aware of the outcomes of the expert panel meetings and potentially could have influenced the focus groups’ discussions. Since, the focus groups were conducted by different facilitator in different contextual circumstances (each participant worked in different clinics), this contributes to different interpretations of the same phenomenon that may enhance trustworthiness of finding (Tong & Craig, 2019).

Additionally, the participants were asked to focus on the provision of post-diagnostic support in a general sense. They were not asked to consider specific groups such as people with young onset dementia, cultural and linguistically diverse communities, or Aboriginal and Torres Strait Islander peoples. The most frequent profession was geriatrician, which may bias the outcomes to people 65 years of age or older who are diagnosed with dementia. Thus, the reported results, while valuable, are not generalisable.

### Future directions

The essential views of people with dementia and their care partners about post-diagnostic care are the focus of the next stage of this study. Understanding the views of those who need ongoing post-diagnostic care and those who provide such care will enable a comprehensive and focused approach to state and national politicians in order to facilitate equitable and continuous post-diagnostic care.

## Conclusion

An analysis of comments from two groups of health professionals showed that all included clinics provide diagnostic assessment. Support at public clinics following diagnosis was limited to ensuring the diagnosis was understood, written information about some post-diagnostic resources was provided, and dementia medications were prescribed. The need for care continuation was met only in private clinics as they provided follow-up by medical specialists, but they also lacked other resources. Support at both public and private memory clinics in regional areas was limited due to fewer available specialists and services. Overall, post-diagnostic support in Australian memory clinics shows an inequity and a substantial difference between metropolitan and regional areas. The focus on regional clinics remains a focus for further development. Recognising ongoing funding constraints as a common problem regardless of geographical locations of clinics, this study calls for government investment. Meanwhile, health professionals need to be encouraged to focus on what is available and feasible, for example vigorous collaboration with primary care along with education for GPs and partnering with community agencies and the third sector.
